# Overexpression of a bHLH1 Transcription Factor of *Pyrus ussuriensis* Confers Enhanced Cold Tolerance and Increases Expression of Stress-Responsive Genes

**DOI:** 10.3389/fpls.2016.00441

**Published:** 2016-04-05

**Authors:** Cong Jin, Xiao-San Huang, Kong-Qing Li, Hao Yin, Lei-Ting Li, Zheng-Hong Yao, Shao-Ling Zhang

**Affiliations:** ^1^State Key Laboratory of Crop Genetics and Germplasm Enhancement, College of Horticulture, Nanjing Agricultural UniversityNanjing, China; ^2^College of Rural Development, Nanjing Agricultural UniversityNanjing, China

**Keywords:** basic helix-loop-helix, cold tolerance, *Pyrus ussuriensis*, reactive oxygen species, *PubHLH1*

## Abstract

The basic helix-loop-helix (bHLH) transcription factors are involved in arrays of physiological and biochemical processes. However, knowledge concerning the functions of bHLHs in cold tolerance remains poorly understood. In this study, a *PubHLH1* gene isolated from *Pyrus ussuriensis* was characterized for its function in cold tolerance. *PubHLH1* was upregulated by cold, salt, and dehydration, with the greatest induction under cold conditions. PubHLH1 had the transactivational activity and localized in the nucleus. Ectopic expression of *PubHLH1* in transgenic tobacco conferred enhanced tolerance to cold stress. The transgenic lines had higher survival rates, higher chlorophyll, higher proline contents, lower electrolyte leakages and MDA when compared with wild type (WT). In addition, transcript levels of eight genes associated with ROS scavenging, regulation, and stress defense were higher in the transgenic plants relative to the WT under the chilling stress. Taken together, these results demonstrated that *PubHLH1* played a key role in cold tolerance and, at least in part, contributed to activation of stress-responsive genes.

## Introduction

Cold stress is regarded as a key environmental factor that impairs plant growth and development, geographic distribution and crop productivity (Chinnusamy et al., 2007; Hu et al., 2013). Therefore, improvement of cold tolerance has been the major subject of intense studies and interest over a long period. As a supplementation for traditional breeding, genetic engineering has been proven to be a powerful strategy for generation of novel germplasms with enhanced cold tolerance. In this regard, it is necessary to characterize valuable genes with function in cold tolerance that can be genetically engineered.

Being sessile organisms, plants have evolved a set of molecular, physiological, and biochemical processes to adapt to the cold stress. During the last decades, enormous studies using a lots of strategies associated with physiology, biochemistry, genomics, and genetics have been implemented to decipher plant responses to cold stress. It is now accepted that cytosolic calcium^+^ influx, inositol phosphates, membrane fluidity, and reactive oxygen species (ROS) play significant roles in altering gene activities, increasing new protective gene product and secondary metabolism to enhance the cold tolerance of plants (Nordin Henriksson and Trewavas, 2003; Los and Murata, 2004; Miura et al., 2005; Suzuki et al., 2012). The noticeable signature is evident change in transcriptional levels of a large number of cold-responsive genes, leading to a large range of physiological and biochemical modifications by which the plants can overcome the cold stress.

The basic helix-loop-helix (bHLH) transcription factors (TFs) are a large gene family in the plant genome (Toledo-Ortiz et al., 2003). There are 167 members in *Arabidopsis* and 162 in rice (Bailey et al., 2003; Li et al., 2006), and they play important roles in transcriptional networks. Some plant bHLH TFs were responsive to abiotic stresses. For example, INDUCER OF CBF EXPRESSION 1 (ICE1) and ICE2 of *Arabidopsis* and *MdCIbHLH1* of *Malus domestica* were involved in the cold signal transduction process (Yamaguchi-Shinozaki and Shinozaki, 2006). *PtrbHLH*, a citrus (*Poncirus trifoliata*) bHLH gene functioned in cold tolerance by positively regulating POD-mediated ROS removal (Huang et al., 2013). Recently, other plant bHLH genes, such as *AtbHLH104* and *CdbHLH1*, were also induced by iron deficiency by a FIT-independent pathway (Zhao et al., 2009; Long et al., 2010). In addition, some plant TFs, for example, AtMYC2, OsbHLH148, and PIF3, were involved in abscisic acid (ABA), jasmonate and photo-induced signal transduction, respectively (Abe et al., 2003; Monte et al., 2004; Seo et al., 2011); *OrbHLH2* of rice (wild rice) improved tolerance to salt and osmotic stress (Zhou et al., 2009); the overexpression of *OrbHLH001*, a rice (wild rice) bHLH gene, conferred freezing and salt tolerance in transgenic *Arabidopsis* (Li et al., 2010); the expression of *OsbHLH148* in rice (*Oryza sativa*) led to drought tolerance by the jasmonate signaling pathway (Seo et al., 2011); ICE1 of *Pyrus ussuriensis* (pear) improved tolerance to cold by enhancing *PuDREBa* transcriptional levels through interacting with PuHHP1 (Huang et al., 2015). Taken together, these results show that plant bHLH TFs are involved in the regulation of plant responses to abiotic stresses.

Although some plant cold-related bHLH TFs have been characterized in *Arabidopsis*, such as ICE1 or ICE2, the functions of these bHLH orthologs in the cold tolerance remain poorly characterized in non-model plants, especially the perennial fruit trees. In addition, knowledge is still little concerning the function of bHLH TFs in cold tolerance. *Pyrus ussuriensis* is a cold-tolerant species, making it a good source to isolate genes of agronomical importance with potential use for genetic engineering. In order to identify and characterize the *Pyrus ussuriensis* homolog of the *Arabidopsis* ICE1-like genes, in this study, we report the molecular cloning and functional characterization of *PubHLH1* isolated from *Pyrus ussuriensis*. *PubHLH1* was revealed to be cold-responsive, and its overexpression in tobacco conferred enhanced resistance to cold and oxidative stresses. Taken together, these data demonstrate that *PubHLH1* plays a positive role in conferring cold tolerance to transgenic plants. It may be an important candidate gene for molecular breeding of cold-tolerance plants.

## Materials and Methods

### Plant Materials and Stress Treatments

Uniform and healthy shoots were collected from 45-day-old *Pyrus ussuriensis* seedlings and subjected to stress treatment. The shoots were grown for 1 day in a growth chamber to minimize the mechanical stress on the tissues, followed by exposure to the corresponding stress treatments, which were carried out as follows. For the cold treatment, seedlings were placed in a growth incubator set at 4°C for 0, 1, 5, 12, 24, and 48 h. Salt stress was carried out by placing the shoots in 200 mM NaCl solution for 0, 1, 5, 12, and 24 h. For dehydration stress, the shoots were put on dry filter papers at 25°C ambient environment for 0, 0.5, 1, 3, and 6 h. For each treatment, at least 60 seedlings were used, and leaves were sampled from three randomly collected seedlings at designated time point and mixed as a material sample pool. Three technical replicates were used for each sample, and the data are shown as means ± standard errors (SE; *n* = 3). Three biological replicates were used for each of the genotypes, the wild type, OE4, OE9. Leaves from all of the treatments were harvested and immediately frozen in liquid nitrogen, and stored at -80°C until further use.

### Cloning and Bioinformatics Analysis of *PubHLH1*

The pear genome database^1^ was searched using as an entry keyword ‘ICE1,’ which yielded two outputs. The first is composed of a complete open reading frame (ORF) which displays 99% sequence identity to *PuICE1* (Huang et al., 2015), while the second includes a total of 13 EST sequences that were merged into one contig with a complete ORF. Based on the contig sequence, a pair of primers (GSP1, Supplementary Table 1) were designed for RT-RCR amplification of *Pyrus ussuriensis* cDNA prepared from seedlings treated for 12 h at 4°C. The PCR mixture in a total 50 μl reaction volume contained 300 ng cDNA, 1x TransStart FastPfu buffer, 0.25 mM deoxyribonucleotide (dNTP), 0.4 μM of each primer and 2.5 units of TransStart FastPfu DNA polymerase. PCR was performed by a program as follows: initial denaturation at 95°C for 2 min, 40 cycles of 95°C for 20 s, 55°C for 20 s, and 72°C for 60 s, followed by a final extension at 72°C for 5 min. Specific PCR products were isolated, subcloned into pMD 18-T vector (Takara, China), and the plasmids were sequenced in Invitrogen (Shanghai, China). Sequence analysis was performed in the NCBI^2^; the theoretical isoelectric point (*pI*) and molecular weight were calculated by ExPASy^3^; phylogenetic tree was constructed by the neighbor-joining method using MEGA4; and analysis of the bHLH domain was carried out on the Motif scan^4^.

### Gene Expression Analysis by Quantitative RT-PCR

The total RNA was extracted from leaves using a cetyltrimethyl ammonium bromide (CTAB)-based method and digested with RNase-free DNase I (Thermo) to remove DNA contamination. Approximately 1 μg of total RNA was reversely transcribed into cDNA using the ReverTra Ace qPCR RT Kit (Toyobo, Shanghai, China) according to the manufacturer’s instructions. Quantitative real-time RT-PCR (qRT-PCR) was used for measuring transcript levels of *PubHLH1* and of stress-related genes. The PCR solution (20 μl) contained 10 μl of SYBR-Green PCR Master Mix (SYBR Premix EX Taq^TM^, TaKaRa), 0.25 μM of each primer (GSP2), 100 ng of cDNA template, and nuclease-free water. QRT-PCR analysis with a SYBR Green PCR kit was performed in a LightCycler 480 (Roche, USA) Real-Time System; the PCR reaction conditions were as follows: 95°C for 5 min, then 45 cycles of 94°C for 10 s, 60°C for 30 s, and 72°C for 30 s, followed by a final extension at 72°C for 3 min. Each sample was analyzed in four replicates, and the 2^-ΔΔCT^ method (Livak and Schmittgen, 2001) was applied to calculate relative expression levels of each gene. *Tubulin* (AB239681) and *Ubiquitin* (DQ830978) was used as an internal control for *Pyrus ussuriensis* and tobacco, respectively, and to normalize the relative expression level of each gene. The expression analysis of each time point was repeated at least four times, and the data were shown as the mean values ± SE.

### Subcellular Localization of PubHLH1

To determine the subcellular localization of PubHLH1, the full-length ORF without the termination codon of PubHLH1 was amplified from pMD 18-T-PubHLH1 using GSP3. The confirmed product was double-digested with *Xba*I and *BamH*I, and then fused to the GFP coding sequence under the control of the CaMV 35S promoter to generate a fusion construct (PubHLH1-GFP). Another fusion construct (AtICE2-GFP) was used as a positive control, which was localized in the nucleus (Fursova et al., 2009). A plasmid containing the GFP gene under control of the *CaMV* 35S promoter was used as a control. Each plasmid was transformed into *Arabidopsis* mesophyll protoplasts using the PEG method (Lee et al., 2003; Agarwal et al., 2006). After incubation for 24 h in darkness, GFP fluorescence was visualized using a confocal microscope (Zeiss LSM 710, Germany). The fusion construct (PubHLH1-GFP) and the control vector (GFP alone) were also separately infiltrated into tobacco epidermal cells; transient transformation of tobacco leaves was done as described by Kumar and Kirti (2010). The transformed leaves were then cultured on MS medium for 2 days before examination of GFP under a confocal microscope. The position of nucleus was revealed by staining with 4′,6-diamidino-2-phenylindole (DAPI).

### Transcriptional Activation Assay of PubHLH1

For the transactivation assay, the full-length ORF of *PubHLH1* was amplified by PCR using primers (GSP5) containing either a *BamH*I or *Xho*I restriction site, and the amplicon was inserted into the same enzyme recognition sites of pENTR3C (Invitrogen). The PubHLH1 fragment was then fused downstream of the yeast GAL4 DNA-binding domain in pDEST32 by recombination reactions (Invitrogen). The fusion vector and the negative control (pDEST32) were independently expressed in yeast strain MaV203 (Invitrogen) according to the manufacturer’s instructions. The transformed yeast strains were placed on SD/–Leu/–Trp or SD/–Leu/–Trp/–His medium with or without different concentrations of 3-amino-1,2,4-triazole (3-AT, 0, 5, and 15 mM) and cultured for 3–4 days to test the expression of the reporter gene *HIS3*.

### Transformation of Tobacco and Regeneration of Transgenic Plants

The specific primers (GSP1) containing the restriction sites *Xho*I and *Kpn*I were used to amplify *PubHLH1* cDNA. The confirmed plasmid was double-digested with *Xho*I and *Kpn*I and then ligated into the pBI121 vector driven by the CaMV 35S promoter (Supplementary Figure S2A). Subsequently, the recombinant plasmid, pBI121*-PubHLH1*, was transferred into the *Agrobacterium* strain GV3101 and used for tobacco (*Nicotiana tabacum*) transformations using leaf disks as explants (Horsch et al., 1985; Huang et al., 2010). The methods of co-culture and selection of explants by kanamycin-resistance were as previously described (Huang et al., 2010). Genomic DNA was extracted from the young leaves of kanamycin-resistant T_0_ transgenic plants and untransformed wild type (WT) using a CTAB-based method. PCR amplifications for the detection of *PubHLH1* were performed with two pairs of primers (*NPTII* and CaMV 35S-*PubHLH1*). Only those yielding the expected PCR fragments by two pairs of primers were considered as positive.

### Cold Tolerance Analysis of the Transgenic Plants

Genomic DNA of T_0_ and T_2_ generation transgenic plants and the WT was extracted using CTAB as extraction buffer. PCR reaction solution was the same as that of RT-PCR except the use of DNA template and primers specific to *PubHLH1* (GSP2, Supplementary Table 1). Ubiquitin was analyzed as internal reference control for tobacco to normalize expression levels (Xian et al., 2014). Overexpression of *PubHLH1* in the selected T_2_ positive transgenic lines was analyzed by semi-quantitative RT-PCR according to Fu et al. (2011) with a slight modification. For cold resistance analysis, the aerial parts of 30 or 60-day-old seedlings of each line were directly exposed to 0°C for 18 h without cold acclimation, following a previously described protocol (Hegedüs et al., 2004) with minor modifications, and then they were allowed to recover under normal conditions for 10 days. For the chilling assay, the survival rate was evaluated after recovery. At the end of the chilling stress, malondialdehyde (MDA), total chlorophyll, electrolyte leakage (EL) and proline (Pro) content; superoxide (O_2_^-^) and hydrogen peroxide (H_2_O_2_) accumulation; cell death; catalase (CAT, EC 1.11.1.6), peroxidase (POD, EC 1.11.1.7), and superoxide dismutase (SOD, EC 1.15.1.1) activities were measured.

### Physiological Measurements and Histochemical Staining

Malondialdehyde contents were measured using the thiobarbituric acid (TBA)-based colorimetric method (Heath and Packer, 1968) with slight modifications. EL was determined based on a previously described protocol (Liu et al., 2006) with minor modifications. Total chlorophyll was extracted and assayed as described previously (Liu et al., 2007; Huang et al., 2010). *In situ* accumulation of H_2_O_2_ and O_2_^-^ was examined based on histochemical staining by nitroblue tetrazolium (NBT) and 3, 3′-diaminobenzidine (DAB), respectively. Details of these assays were described by Huang et al. (2010) and Shi et al. (2010). The cell death was detected with trypan blue staining based on the method of Pogany et al. (2009) and Huang et al. (2011). Activities of CAT, POD, and SOD were analyzed using the detection kits (Nanjing Jiancheng Bioengineering Institute, China) based on the manufacturer’s instructions, and expressed as Unit/g of fresh weight (FW) or dry weight (DW).

### Statistical Analysis

Cold treatments of the WT and transgenic lines were repeated at least twice. All experimental data are averages of at least three independent replicates, shown as mean ± standard error (SE). The data were analyzed using SPSS software and statistical differences were compared based on Duncan’s multiple range tests.

## Results

### Cloning and Bioinformatics Analysis of *PubHLH1*

**FIGURE 1:**
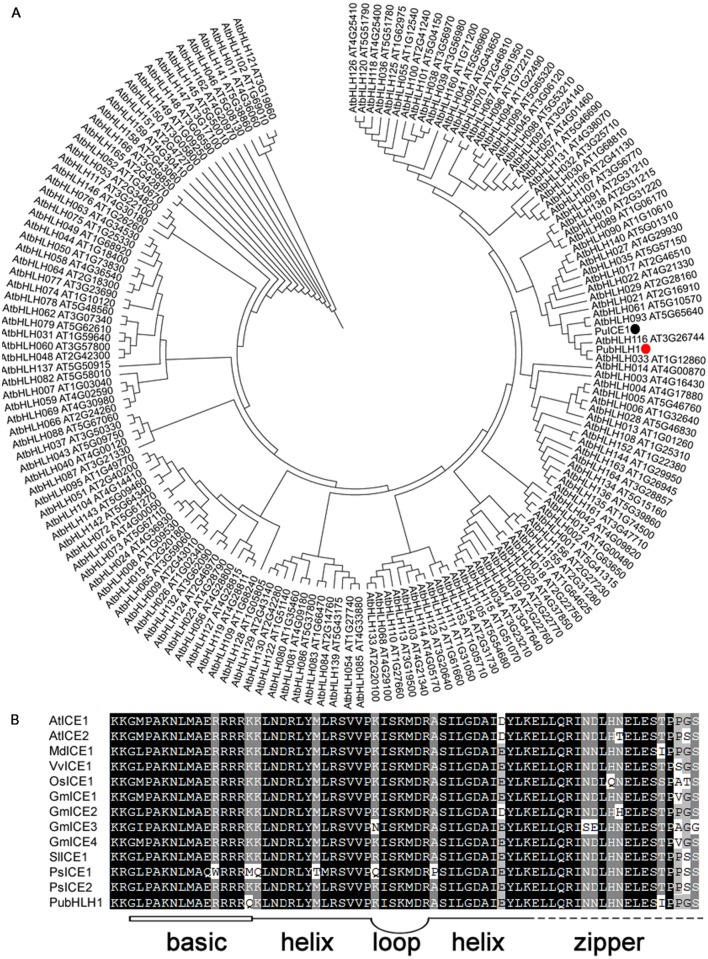
**Phylogenetic tree construction and motif analysis of the conserved bHLH domain. (A)** Phylogenetic tree containing PubHLH1 and *Arabidopsis* bHLHs. PubHLH1 is marked with a red circle. **(B)** Motif analysis of bHLH domains from PubHLH1 and those of *Arabidopsis thaliana* (AtICE1: AT3G26744; AtICE2: AT1G12860), *Glycine max* (GmICE1: ACJ39211; GmICE2: ACJ39212; GmICE3: ACJ39213; GmICE4: ACJ39214), *Malus* × *domestica* (MdICE1: ABS50251), *Oryza sativa* (OsICE1: NP_001045272), *Populus suaveolens* (PsICE1: ABF48720; PsICE2: ADK91821), *Solanum lycopersicum* (SlICE1: AGG38826), and *Vitis vinifera* (VvICE1: AFI49627) were performed on MEME. The basic region (15 amino acids) is shown as an open box, and helix-loop-helix domain is shown by bold lines linked by a loop. The broken line indicates the zipper region following the bHLH domain.

Increasing evidence shows that plant bHLH TFs play an important role in abiotic stress responses. So, we used a method that combined bioinformatics database search and RT-PCR to isolate bHLH-like genes. RT-PCR of *Pyrus ussuriensis* cDNA was carried out with a pair of primers designed according to the contig merged from the retrieved sequences, and produced 1921 bp fragment which was verified by sequencing. The putative gene was thus designated as PubHLH1 and deposited in GenBank with the accession number KU358904. PubHLH1 gene encodes a polypeptide of 545 amino acids with a calculated molecular mass of 59 kDa and an isoelectric point of 6.05. The phylogenetic tree constructed based on the sequences of PubHLH1 and the *Arabidopsis* bHLHs indicates that PubHLH1 is most closely related to AtbHLH33 (**Figure 1A**). Motif scanning demonstrated that PubHLH1 protein sequence included a typical bHLH domain of 48 amino acids, comprising a basic region of 15 amino acids and two helices (14 amino acids each) that were connected by a five amino acid loop. The bHLH domain of PubHLH1 showed a high degree of identity with those from other plant species (**Figure 1B**).

### Expression Patterns of *PubHLH1* under Abiotic Stresses

**FIGURE 2:**
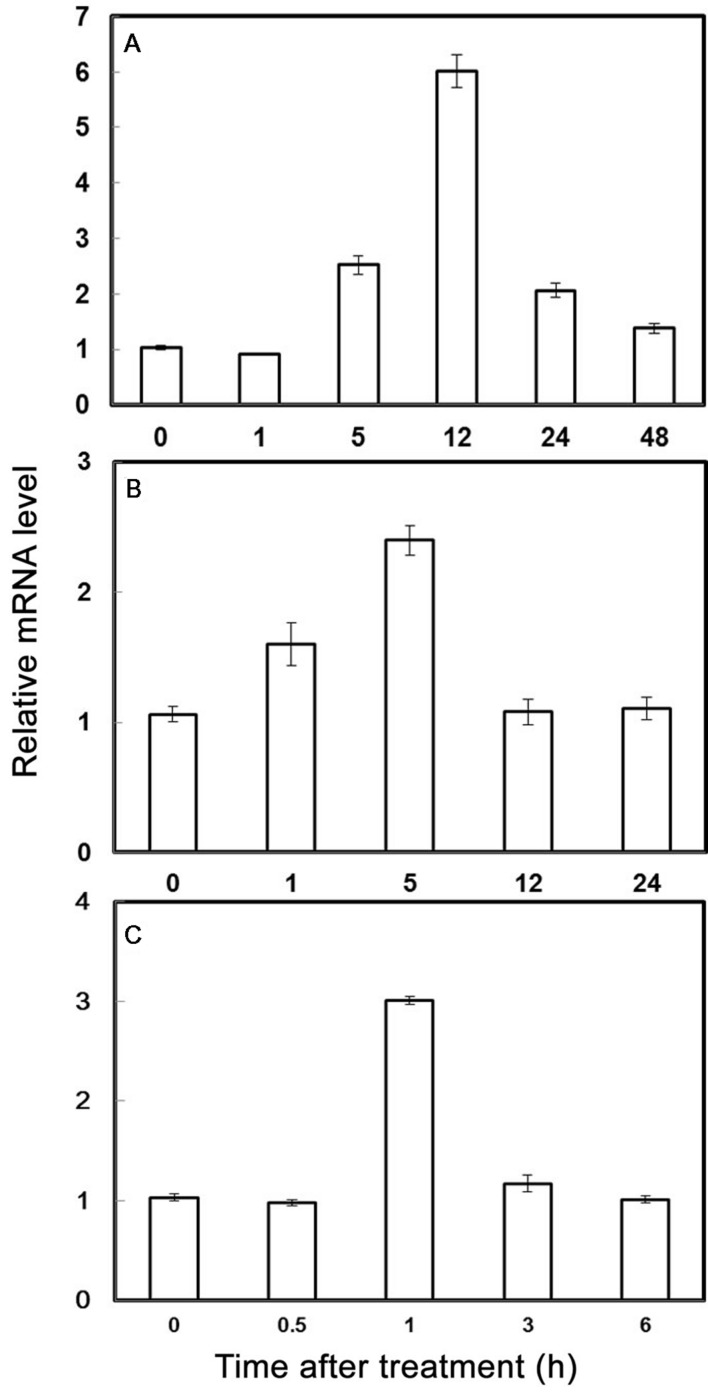
**Time-course expression levels of *PubHLH1* in *Pyrus ussuriensis* under the cold (A), salt (B), and dehydration (C) treatments.** Data represent the means ± SE of four replicates.

The expression of *PubHLH1* under various treatments including cold, salt, and dehydration, was analyzed by qRT-PCR. As showed in **Figure 2**, the transcript level of *PubHLH1* exhibited a slight decrease at 1 h under cold stress, and then elevated progressively until reaching the peak value at 12 h (greater than 6.5 times induction), followed by a noticeable decrease at the last time point (**Figure 2A**). Under salt treatment, *PubHLH1* transcripts showed steady elevation within 5 h, but greatly decreased to the last time point (**Figure 2B**). Upon exposure to dehydration treatment, *PubHLH1* was induced to peak value at 1 h, but then decreased to its original level in the remaining time points (**Figure 2C**). In conclusion, the cold treatment caused a stronger induction of *PubHLH1* transcript level compared with salt and dehydration, indicating that *PubHLH1* might play important roles against the abiotic stresses, particularly in cold.

### Subcellular Localization of PubHLH1

**FIGURE 3:**
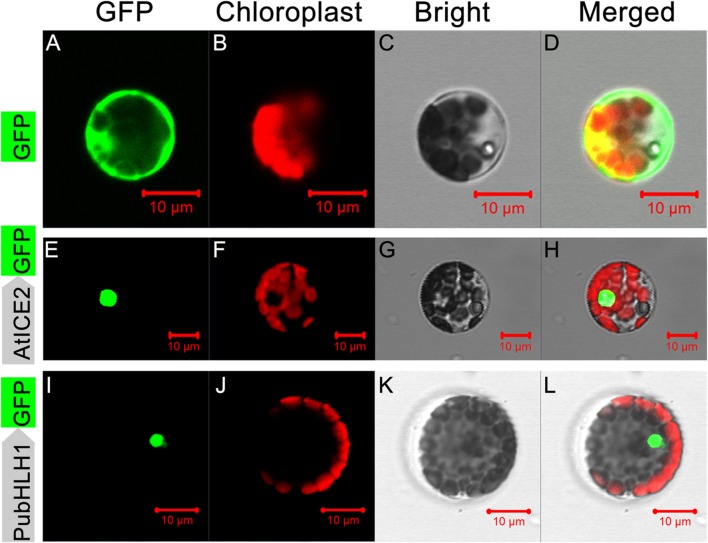
**Subcellular localization of *PubHLH1* in *Arabidopsis* protoplasts.** *Arabidopsis* protoplasts were transformed with constructs containing fusion plasmid (PubHLH1:GFP, **I–L**), positive control (AtICE2:GFP, **E–H**) or control (GFP alone, **A–D**). Images under dark field (left), bright field **(C,G,K)** and merged images **(D,H,L)**.

Sequence analysis showed that PubHLH1 contains a nuclear localization signal, implying that it may be localized to the nucleus. To test this, the ORF of PubHLH1 was fused to N-terminus of the GFP protein under the control of the CaMV 35S promoter. The fusion protein (PubHLH1-GFP), a positive control (AtICE2-GFP), and control (GFP alone) were separately transformed into *Arabidopsis* protoplast cells. Microscope observation showed that the green fluorescence was distributed in the whole cells when the control plasmid was used, whereas green fluorescence was detected only in the nuclei when the vectors contained PubHLH1-GFP or the positive control (AtICE2-GFP; **Figure 3**). Nuclear localization of PubHLH1 was further analyzed in tobacco leaf epidermis via *Agrobacterium-mediated* transformation (Supplementary Figure S1). Microscopic visualization showed that the control GFP was uniformly distributed throughout the whole cell (Supplementary Figures S1A–D), whereas the PubHLH1-GFP fusion protein was observed exclusively in the nucleus (Supplementary Figures S1E–H). These results suggest that PubHLH1 is a nuclear protein.

### PubHLH1 Has Transactivation Activity in Yeast

**FIGURE 4:**
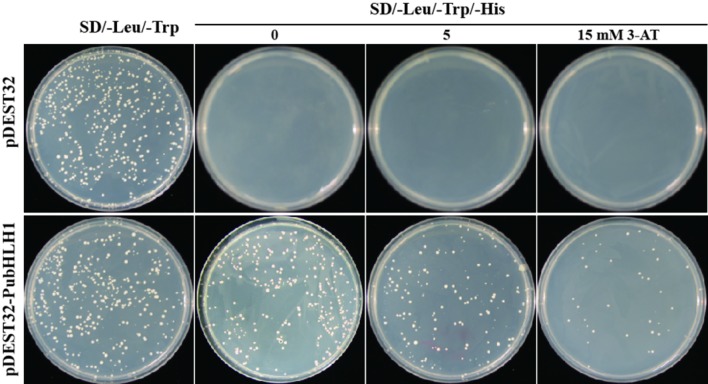
**Transcriptional activation assay of *PubHLH1* in yeast.** Growth of yeast cells (MaV203) transformed with either control vector **(Upper)** or the fusion vector harboring *PubHLH1* **(Under)** on SD/–Leu/–Trp or SD/–Leu/–Trp/–His supplemented with 0, 5, or 15 mM 3-AT.

The Y2H (yeast two-hybrid) system was used to determine whether PubHLH1 functioned as a transcriptional activator in yeast. For this purpose, the PubHLH1 ORF was fused in frame downstream of the yeast GAL4 DNA-binding domain of vector pDEST32 (Invitrogen). The fused vector was transformed into yeast MaV203 strain (Invitrogen) according to the manufacturer’s instructions. The transformed yeast cells carrying the control plasmid grew well on the SD/–Leu/–Trp medium, but failed to grow on SD/–Leu/–His/–Trp medium. Yeast cells transformed with the fusion plasmid also grew on the SD/–Leu/–Trp/–His medium with 5 mM 3-AT, and even with 15 mM 3-AT (**Figure 4**), indicating that PubHLH1 had transcriptional activity in yeast cells.

### Overexpressing *PubHLH1* Enhances Tolerance to Cold

To investigate the function of *PubHLH1*, transgenic tobacco plants overexpressing *PubHLH1* were generated via *Agrobacterium*-mediated tobacco leaf disks transformation. Totally, seven T_0_ lines were confirmed by PCR with primers specific to CaMV 35S and *NPTII*, four out of them were further confirmed as transgenic plants by PCR with specific primers of *PubHLH1* (Supplementary Figures S2B,C), and transcript levels of *PubHLH1* were higher in two lines (OE4 and OE9) as was verified by semi-quantitative RT-PCR analysis (Supplementary Figure S2D). To ensure the accuracy of the experiment, the transgene overexpression in the T_2_ lines was further analyzed by RT-PCR, which indicated that PubHLH1 expressed similarly in different individuals from T_2_ transgenic lines (Supplementary Figure S2E).

**FIGURE 5:**
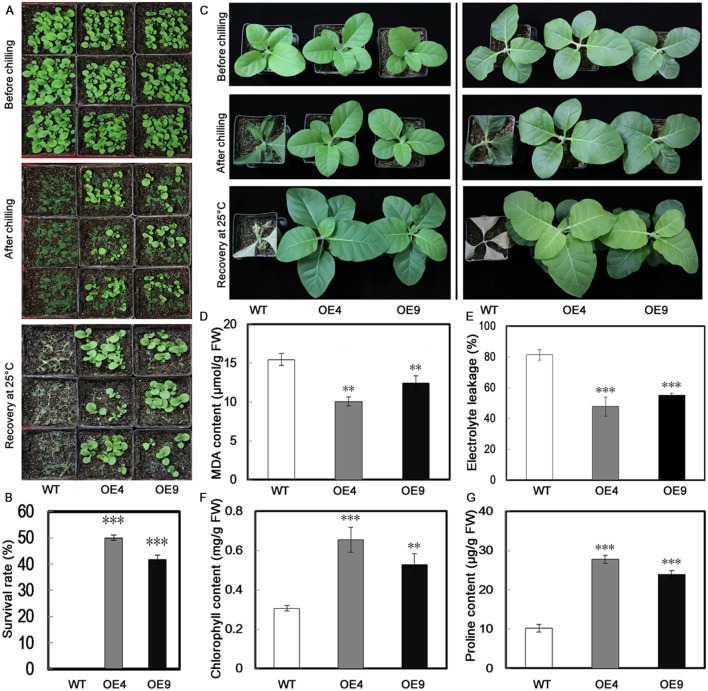
**Overexpression of *PubHLH1* enhances cold tolerance in transgenic tobacco. (A)** Phenotypes of 30-day-old seedlings of WT and transgenic plants (OE4 and OE9) subjected to chilling treatment (0°C) for 18 h, followed by recovery at the normal growth temperature for 10 days. **(B)** Survival rates of the WT and transgenic plants after recovery from chilling stress. The survival rate is the ratio of the number of living plants after recovery from the chilling treatment to the total number of plants tested. **(C)** Phenotypes of 60-day-old WT and transgenic plants (OE4 and OE9) before and after chilling stress, followed by recovery for 15 days. **(D–G)** MDA content, electrolyte leakage, chlorophyll, and proline contents, respectively. Fresh weight (FW). Data represent the means ± SE of at least three replicates. Significant differences between WT and transgenic plants are indicated by asterisks (^∗∗^*P* < 0.01, ^∗∗∗^*P* < 0.001).

To evaluate the function of *PubHLH1* in cold tolerance, T_2_ transgenic tobacco plants and the WT were subjected to cold treatment at chilling temperature (0°C) using 30-day-old seedlings. Under normal growth conditions, no morphological differences were noticed between the transgenic lines and WT (**Figure 5A**). When seedlings were exposed to chilling stress (0°C) for 18 h, more serious chilling injuries were seen in WT plants, but transgenic plants were affected to a lesser extent. After recovery for 10 days under normal conditions, all WT plants were dead (**Figure 5A**), but the survival rates of OE4 and OE9 were 50 and 41.67%, respectively (**Figure 5B**).

When the 60-day-old plants were treated for 18 h at 0°C, the similar phenotypes were obtained: the WT plants exhibited more damage, whereas transgenic plants were less affected (**Figure 5C**). MDA and EL, important indicators of cell injuries, can reflect the extent of damage of the membranes. The assay showed that MDA content in the two transgenic lines was lower than that of the WT (**Figure 5D**). We compared the EL of WT and the transgenic lines after cold treatment. As shown is **Figure 5E**, the EL of WT was 81.4%, while EL of the two transgenic lines was significantly lower, 47.8% for OE4 and 55.2% for OE9, showing that membrane damage was alleviated in the transgenic lines. Chlorophyll level, a parameter indicating leaf damage associated with cold stress, was also quantitatively measured. After the chilling treatment, higher chlorophyll content was measured in the leaves of transgenic lines than in the WT (**Figure 5F**). We also measured proline contents in the transgenic lines and WT after chilling treatment, as this compound is considered as an important metabolite indicating the relevance to cold tolerance. As shown in **Figure 5G**, the transgenic lines accumulated more proline in comparison with the WT.

It would be interesting to compare the expression of the tobacco gene *NtbHLH1*, the closest homolog of *PubHLH1*, with the expression of *PubHLH1* in transgenic lines. Therefore, we analyzed their transcript levels in both WT and transgenic plants under both control and chilling stress (Supplementary Figure S3). When the plants were exposed to chilling stress (0°C for 16 h), the relative mRNA level of *NtbHLH1* in WT was fourfold, slightly lower than that of the transgenic lines: 4.27-fold for OE4 and 4.16-fold for OE9. These results indicate that the transgenic tobacco plants were more tolerant to cold stress, mainly through the overexpression of *PubHLH1* in tobacco. In the future, more work is required to experimentally clarify different transcript levels between *PubHLH1* and *NtbHLH1* in the transgenic lines, and to decipher their role in cold tolerance.

### Analysis of Cell Death and ROS Levels by Histochemical Staining

**FIGURE 6:**
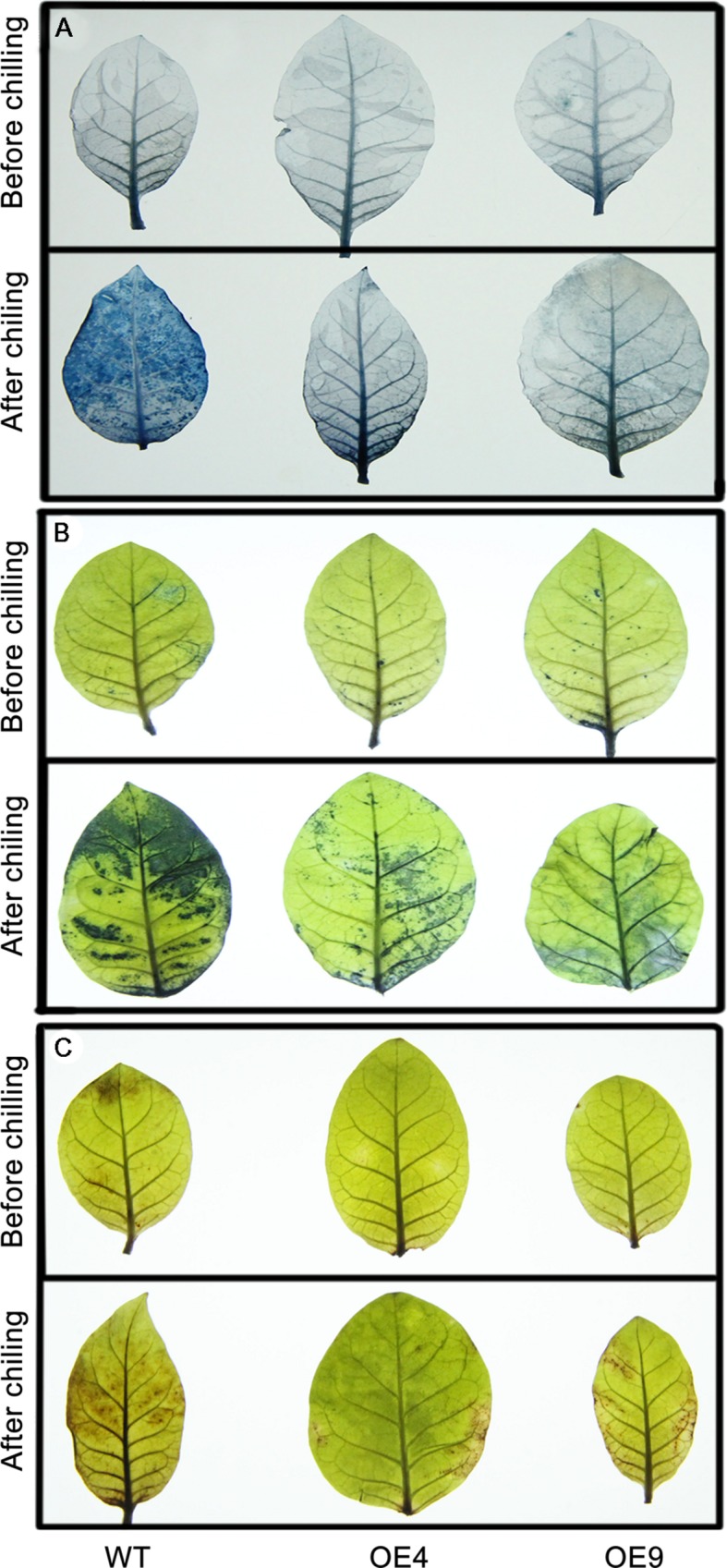
**Histochemical staining via trypan blue, nitro blue tetrazolium (NBT), and 3,3′-diaminobenzidine (DAB) to analyze cell death, and the accumulation of O_2_^-^ and H_2_O_2_ in the transgenic (OE4 and OE9) and WT plants. (A)** Representative photographs show the staining of cell death in the leaves before (upper panel) and after the chilling treatment. **(B,C)** The *in situ* accumulation of O_2_^-^ and H_2_O_2_ in the transgenic and WT plants before and after the chilling stress.

Trypan blue staining was used to reveal cell death after the chilling treatment. As shown in **Figure 6A**, the WT leaves were stained to a larger extent relative to the transgenic lines OE4 and OE9, suggesting that they experienced more detrimental damage. Abiotic stresses result in the excessive accumulation of ROS, such as O_2_^-^ or H_2_O_2_, so we determined ROS levels in the tested samples. The leaves of WT and transgenic lines that have been exposed to chilling were stained with NBT and DAB to assess *in situ* accumulation of O_2_^-^ or H_2_O_2,_ respectively. Before the chilling treatment, ROS accumulation of the transgenic lines and the WT plants was similar as their leaves were equivalently stained. However, after the chilling treatment, the two transgenic plants were stained to a lower degree by both NBT (**Figure 6B**) and DAB (**Figure 6C**) when compared with WT plants, indicating that overexpressing lines accumulated less ROS than the WT upon chilling treatment.

### Antioxidant Enzyme Activities and Stress-Related Gene Expression Levels in Transgenic Lines

**FIGURE 7:**
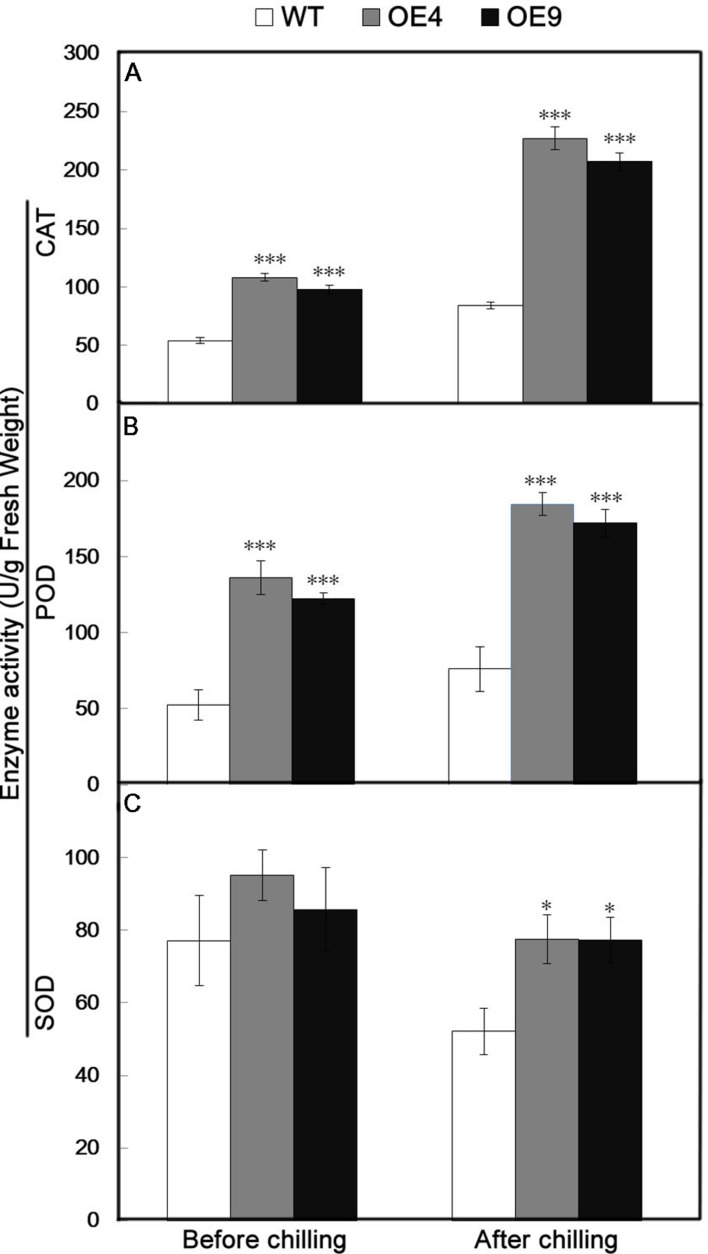
**Analysis of antioxidant enzyme activity in the WT and two transgenic lines (OE4 and OE9) before and after chilling treatment. (A–C)** Activities of CAT, POD, and SOD, respectively. Data represent the means ± SE of at least three replicates. The significant differences between the WT and transgenic plants are indicateded by asterisks (^∗^*P* < 0.05, ^∗∗∗^*P* < 0.001).

Antioxidant enzymes, such as CAT, POD, and SOD, play significant roles in ROS scavenging and thus influence the cellular ROS level. Since the two overexpressing lines contained fewer ROS relative to the WT, the activity of three key antioxidant enzymes (CAT, POD, and SOD) were assessed in the leaves sampled before and after chilling stress. The activities of these enzymes in transgenic lines were higher than in WT under normal conditions (**Figure 7**). After the chilling treatment, CAT and POD activities were notably enhanced in the transgenic lines, and a slight increase occurred in the WT (**Figures 7A,B**). Exposure to the chilling treatment resulted in a slight decrease in the SOD activity of all of the tested lines, but it was still significantly higher in the transgenic lines than in the WT (**Figure 7C**).

**FIGURE 8:**
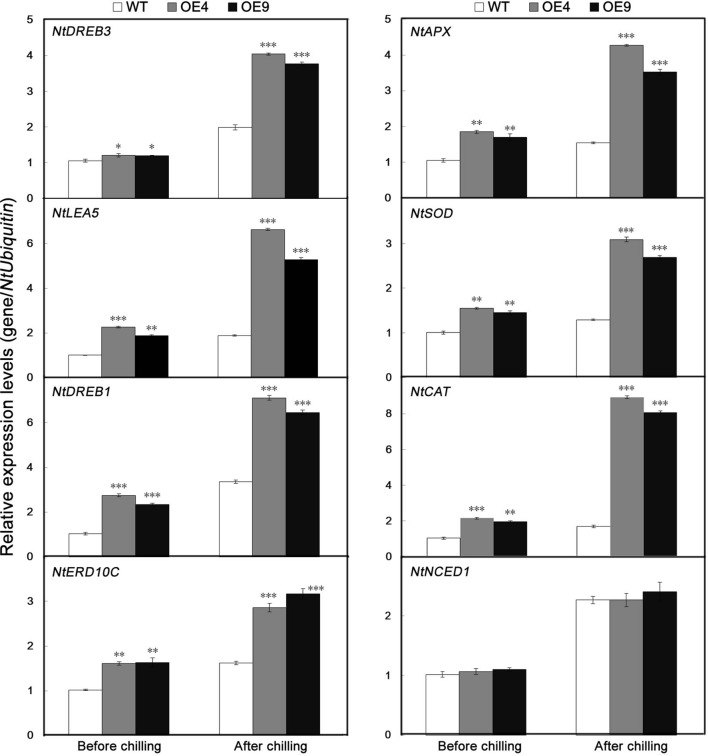
**Analysis of relative expression levels of stress-responsive and ROS-related genes by quantitative real-time PCR in WT and transgenic lines (OE4 and OE9) before and after chilling treatment.** Data represent the means ± SE of at least three replicates. Significant differences between the WT and transgenic plants are indicated by asterisks (^∗^*P* < 0.05, ^∗∗^*P* < 0.01, ^∗∗∗^*P* < 0.001).

To gain further insight into the molecular mechanism underlying the enhanced cold resistance in the *PubHLH1*-overexpressing lines, the expression patterns of eight ROS-related or stress-responsive genes was examined in the WT and transgenic lines before and after chilling treatment (**Figure 8**). These genes included three ROS detoxification (*NtAPX*, *NtSO*D, and *NtCAT*), two significant cold stress regulatory proteins (*NtDREB1* and *NtDREB3*) and three stress defensive proteins (*NtLEA5*, *NtERD10C*, and *NtNCED1*). Before the chilling treatment, steady state mRNA levels of eight genes in two overexpressing lines were higher than those in the WT. After the chilling stress, the transcript levels of the analyzed genes were slightly upregulated in the WT with the exception of a rapid increase of *NtNCED1*, but more pronouncedly in the two overexpressing lines. So, transcript levels of the analyzed genes were significantly higher in the transgenic lines than in the WT (**Figure 8**). However, there was no difference in the expression of *NtNCED1* between cold-stressed transgenic and WT plants. These results showed that overexpression of *PubHLH1* in tobacco led to change in the transcript levels of endogenous ROS-related and stress-responsive genes before and after chilling treatment.

## Discussion

Numerous studies showed that TFs constitutes the key regulon that play significant roles in regulating various biological processes, including abiotic stress response, by interacting with *cis*-acting elements in their promoter region. Plants have a wide range of TFs, among these, members of NAC, MYB, bZIP, WRKY, AP2/ERF, CBF function in response to environmental stress and improve stress tolerance (Jaglo-Ottosen et al., 1998; Jakoby et al., 2002; Zhu et al., 2005; Hu et al., 2006; Zhang et al., 2009; Huang et al., 2010; Ren et al., 2010; Sun et al., 2014). In contrast, an important TF group, bHLH proteins, have been less well characterized. To date, only a few plant bHLH proteins have been shown to function in the transcriptional regulation of a diversity of abiotic stress (Fursova et al., 2009; Zhou et al., 2009; Li et al., 2010; Seo et al., 2011; Huang et al., 2013). Thus, characterization of more stress-responsive bHLH genes is crucial to decipher the cold signaling pathway pertinent to freezing tolerance and to provide a valuable gene candidates for genetic manipulation.

In this study, we report the identification of a bHLH1 gene in *Pyrus ussuriensis*. Although 177 and 167 bHLHs have been unraveled in the genomes of rice and *Arabidopsis*, little is known about the exact number of bHLH genes in *Pyrus ussuriensis*. A phylogenetic tree constructed based on the sequences of PubHLH1 and bHLHs from *Arabidopsis* revealed that PubHLH1 was most closely related to AtbHLH033 (AtICE2). Motif scanning suggested that the PubHLH1 contains a MYC-like bHLH domain and displays a significant degree of identity with those from other plant species indicating that *PubHLH1* is a putative ICE2 homolog in *Pyrus ussuriensis*.

An important feature of plant bHLHs is the induction of their transcript levels by abiotic stresses (Yuan et al., 2008; Seo et al., 2011; Xie et al., 2012; Huang et al., 2013). For instance, the transcript level of *PtrbHLH033* gene is up-regulated by cold, dehydration, and salt treatment (Huang et al., 2013). The transcript level of *OsbHLH148* is up-regulated at the early stage of drought and salt stress and shows a steady increase under cold stress (Seo et al., 2011). The *OrbHLH2* transcript level is induced by salt and osmotic stress, but not by cold stress (Zhou et al., 2009). In our work, the *PubHLH1* transcript level was transiently induced by salt and dehydration, while the strongest induction of *PubHLH1* transcripts was by cold stress. The expression patterns of the *PubHLH1* were similar to those of *OsbHLH148* (Seo et al., 2011). Although several bHLH TFs were up-regulated by cold, the transcript abundance of *AtICE1* in *Arabidopsis* was not changed by dehydration (Chinnusamy et al., 2003). These data seem to show that the bHLH TFs are diversely modulated under various abiotic stresses, indicating that they possibly play different roles in regulating the response to specific stresses. One explanation is that different members of the same TF family display varying responses because they are regulated by different regulators upstream in the stress signaling network (Wang et al., 2003).

Compared with dehydration and salt, low temperature stress caused stronger induction of *PubHLH1* mRNA abundance, which forced us to do in-depth work to elucidate its function in cold tolerance. To this end, *PubHLH1* was transformed into tobacco via Agrobacterium-mediated transformation under the control of CaMV 35S promoter. The stress tolerance assay demonstrated that the two selected transgenic lines exhibited improved tolerance to chilling stress as compared with the WT, as measured by EL, survival rate, MDA, chlorophyll, and proline contents, along with phenotypic observation, suggesting that overexpression of *PubHLH1* conferred tolerance to cold stress. Our results agreed with earlier reports, in which overexpression of bHLH genes has been shown to render tolerance to abiotic stresses in the transgenic plants (Colangelo and Guerinot, 2004; Feng et al., 2012; Huang et al., 2013), showing that bHLH genes might hold great potential for genetic engineering to improve stress tolerance.

Besides assessing the improved cold tolerance of tobacco transgenics, we carried out several physiological, molecular and biochemical analysis in order to identify the mechanism underling the improved tolerance. We made efforts to carry out more work to find out physiological difference between the transgenic plants and WT under cold stress. Previous studies showed that following the exposure to abiotic stresses, the oxidative burst is the first biochemical response of plants (Joo et al., 2005). We put special emphasis on comparing their ROS levels because it has been widely accepted that in biological systems ROS accumulation is related to physiological perturbation, and ROS levels can reflect the degree of damage to cellular components (Mittler, 2002; Suzuki et al., 2012). As the sub-products of the aerobic metabolism, O_2_^-^ and H_2_O_2_ are two major forms of ROS (Bolwell et al., 1999) and, moreover, ROS overproduction results in an increasing spread of cell death (Torres et al., 2005). MDA, EL, and Pro content levels are related to the membrane system (Foyer et al., 1994), and in our work, a lower EL and MDA level and less extensive cell death in the two transgenic lines imply that they might be subjected to less serious oxidative stress than the WT. Thus, it was of interest to determine the ROS accumulation in the tested lines after chilling stress by histochemical staining. Transgenic lines exhibited clearly less intense DAB and NBT staining in comparison with the WT. However, ROS accumulation greatly relies on the homeostasis between generation and scavenging (Miller et al., 2010). The lower ROS levels in the transgenic lines seem to indicate that they had more effective ROS-scavenging systems compared with WT, and three ROS-scavenging enzymes played a positive role in this process. Of the enzymes, SOD catalyzes the dismutation of O_2_^-^ to H_2_O_2_ and oxygen, which is eliminated by the coordinated actions of POD and CAT (Blokhina et al., 2003). In our study, the activities of CAT, POD, and SOD were slightly higher in the two transgenic lines than in WT under normal conditions. However, when exposed to cold stress, the activities of three enzymes in the transgenic lines were significantly higher than in the WT. This demonstrated that there was a more effective detoxifying system in the two transgenic lines to scavenge redundant ROS during stress and maintain the ROS balance. Thus, a better ROS-scavenging system could protect plants against more damage, and this may be an integral part of cold tolerance in the transgenic plants overexpressing *PubHLH1*.

To further elucidate the molecular mechanisms of *PubHLH1* in cold stress signal perception, the expression levels of stress-responsive and ROS-related genes were analyzed. These genes included three ROS detoxification (*NtAPX*, *NtSO*D, and *NtCAT*), two significant cold stress regulatory proteins (*NtDREB1* and *NtDREB3*) and three stress defensive proteins (*NtLEA5*, *NtERD10C*, and *NtNCED1*), which or whose homologs in other plants have been shown to be involved in abiotic stress response. For example, previous study indicates that C-repeat binding TF (CBFs) and dehydration-responsive element-binding proteins (DREBs) play vital roles in regulating cold stress responses. The overexpression of *DREB/CBF* genes confers cold tolerance in a variety of plants (Maruyama et al., 2004; Qin et al., 2004; Kang et al., 2013). In our study, the expression levels of *NtDREB1* and *NtDREB3* were all higher in the two transgenic lines than in the WT, indicating that the transcriptional levels of the *DREB* genes were induced by the overexpression of *PubHLH1*. Accumulating evidence implies that late embryogenesis abundant (LEA) proteins could function in protecting cells from cold stress (Hwang et al., 2005). Here, as LEA genes, *NtERD10C* and *NtLEA5* encode a group 2 and 5 LEA protein, respectively, and their mRNA levels were higher in the two transgenic lines than in the WT. These results suggested that more LEA proteins are synthesized in the transgenic plants to protect the membrane system from more damage. It was also found that transcript levels of the genes encoding ROS-scavenging enzymes (*NtAPX*, *NtSO*D, and *NtCAT*) were up-regulated in the *PubHLH1*-overexpressing lines before and after chilling stress, consistent with the greater activity of these antioxidant enzymes. This may presumably explain the activation of the antioxidant enzymes in the transgenic lines. It is well known that *NCED* gene encodes a rate-limiting enzyme for ABA synthesis, and ABA plays an essential role in the regulation of stress-responsive genes in plant adaptation to abiotic stresses. In our study, it is interesting that the *NtNCED1* was induced to higher levels both in the transgenic lines and WT after chilling stress. But the difference in the transcript levels of *NtNCED1* between the transgenic lines and the WT was not statistically significant, which showed that *NCED1* may be not a downstream gene of *PubHLH1*. Taken together, these genes may act as the intermediates between *PubHLH1* and the aforementioned functional genes. In this case, *PubHLH1* might function to facilitate transcriptional up-regulation of these endogenous regulatory genes, which in turn activated their downstream target genes, including those mentioned above. In the future, it will be of paramount significance to identify interacting genes located downstream of *PubHLH1*, which will shed light on the molecular mechanism of action underlying the *PubHLH1*-mediated cold tolerance. Moreover, in our work, it is worth mentioning that the transcript level of *PubHLH1* is induced by cold stress under the control of 35S promoter of cauliflower mosaic virus (CaMV35S; Supplementary Figure S3). A possible explanation for this is that *PubHLH1* undergoes certain unidentified modifications upon exposure to cold, for example, *PubHLH1* is the target of a cold down-regulated miRNA, which degrades its transcript. Our hypotheses corroborate previous studies indicating that CdICE1-overexpressing plants experienced significant reduction in miR398 (Chen et al., 2013). Another explanation is that 35S promoter is regulatable and can be induced by some stress, which is in agreement with previous report (Czechowski et al., 2005; Wang et al., 2010). In the future, more work is required to experimentally clarify the reason of the rapid increase of *PubHLH1* transcript level under the control of constitutive promoter 35S after cold stress.

## Author Contributions

X-SH, CJ contributed to the experimental design and management, data analysis, and manuscript preparation. K-QL contributed to proofreading and critical review of this manuscript. CJ, HY, L-TL, and Z-HY contributed to genes expression analysis. X-SH, CJ, and S-LZ designed and managed the experiments. All authors have read and approved the final manuscript.

## Conflict of Interest Statement

The authors declare that the research was conducted in the absence of any commercial or financial relationships that could be construed as a potential conflict of interest.

## Footnotes

1 http://peargenome.njau.edu.cn/

2 http://www.ncbi.nlm.nih.gov/

3 http://www.expasy.org/tools

4 http://myhits.isb-sib.ch/cgi-bin/motif_scan
