# Supplementary material for: Overexpression of a bHLH1 Transcription Factor of Pyrus ussuriensis Confers Enhanced Cold Tolerance and Increases Expression of Stress-Responsive Genes
Source: Front Plant Sci. 2016 Apr 5;7:441. doi: 10.3389/fpls.2016.00441 (PMC4820633; doi:10.3389/fpls.2016.00441)
Supplement: Supplementary file 1 [file Presentation_1.PDF]

Supplemental Data

Supplemental Figure S1. Subcellular localization of PubHLH1. Tobacco epidermal cells were transiently transformed with either control (GFP alone, A-D) or fusion plasmid (PubHLH1: GFP, E-H). Images under fluorescence (A, E), bright field (B, F) and merged images (D, H) were shown. (C) and (G) DAPI staining of the cells in (A) and (E).

.

Supplemental Figure S2. Generation and characterization of *PubHLH1* gene in transgenic tobacco. (A) illustrative diagram of the *PubHLH1* overexpression construct used for tobacco transformation. LB, left border; Tnos, nopaline synthase (nos) terminator; *NPTII*, neomycin phosphotransferase II; 35S, cauliflower mosaic virus 35S promoter; RB, right border. (B and C) PCR identification of kanamycin-resistant T_0 generation tobacco plants using *NPTII*-specific primers and CaMV35S–*PubHLH1* primers. WT, wild type; Lane P, positive control (plasmid PBI121–*PubHLH1*); Lane M, DNA marker; the numbers show different transgenic lines (lines 4 and 9 are designated OE4 and OE9, respectively). (D) Overexpression of *PubHLH1* in T_0 generation transgeinc lines (4, 6, 9 and 15) was verified by semi-quantitative RT-PCR analysis. (E) Analysis of *PubHLH1* gene overexpression in two T_2 generation transgenic lines by RT-PCR. The *Ubiquitin* gene was used as an internal control.

Supplemental Figure S3. Expression levels comparison of *PubHLH1* and *NtbHLH1*

under chilling stress, analysed by qRT-PCR. Transcript levels comparison of *NtbHLH1* and *PubHLH1* in tobacco WT and transgenic lines before (left) and after (right) chilling stress. Asterisks indicate a significant difference between transcript levels of *NtbHLH1* and *PubHLH1* in transgenic lines at the same time point. (***P<0.001).

Supplemental table 1 Primer sequences used for cloning, subcellular localization, vector construction, transgenic confirmation and expression analysis

**Genes**	**Primers**	**Sequences (5′-3′)**	
		**Forward**	**Reverse**
PubHLH1	GSP1	ACCAGCTGAGCTTCTCCTGCAAATCC	CGACACAATTGAGCTATCAGCTATGGC
PubHLH1	GSP2	CCCTTGAGTCCTTCCCTTCT	CCCATCTCCCTCTTCCTCTC
TUB-b2	Tublin	TGGGCTTTGCTCCTCTTAC	CCTTCGTGCTCATCTTACC
PubHLH1	GSP3	TCTAGAATGCTGCCGAGGCTGAACGGT (*Xba* I site is underlined)	GGATCCCACCATGCCATGGAACCCGATC (*BamH* I site is underlined)
PubHLH1	GSP4	GAAGATCTATGCTGCCGAGGCTGAACGGT(*Bgl* II site is underlined)	GACTAGTCACCATGCCATGGAACCCGATC (*Spe*I site is underlined)
PubHLH1	GSP5	GGATCCATGCTGCCGAGGCTGAACGGT (*BamH*I site is underlined)	CTCGAGCACCATGCCATGGAACCCGATC (*Xho*I site is underlined)
35S-PubHLH1		CGCCGTAAAGACTGGCGAACAGTTCATACAGAGT	CGACACAATTGAGCTATCAGCTATGGC
NPTII	NPTII	AGACAATCGGCTGCTCTGAT	TCATTTCGAACCCCAGAGTC
NtDREB1		GGCTGAGATAAATGATACGAC	CATCTCCGTCAAAGTCATAGT
NtDREB3		GCCGGAATACACAGGAGAAG	CCAATTTGGGAACACTGAGG
NtLEA5		CTCTAACTCCAAACTCATCTCTGC	CAAAACCCCAGATTCAAGAC
NtRD29A		AGCTGATCCGGAGAAGAGAATAAC	CATCGGTGCACCCCAATAGT
NtCAT		AGGTACCGCTCATTCACACC	AAGCAAGCTTTGACCCAGA
NtAPX		CAAATGTAAGAGGAAACTCAGAGGA	AGCAACAACTCCAGCTAATTGATAG
NtSOD		GGTGTTTCCAGTGGCGGACG	TCCTCCCCTCAGCTACGGGGTAT
Ubiquitin		AGCTACATGACGCCATTTCC	CCCTGTAAAGCAGCACCTTC
