# Supplementary figures and images for: Overexpression of a bHLH1 Transcription Factor of Pyrus ussuriensis Confers Enhanced Cold Tolerance and Increases Expression of Stress-Responsive Genes
Source: Front Plant Sci. 2016 Apr 5;7:441. doi: 10.3389/fpls.2016.00441 (PMC4820633; doi:10.3389/fpls.2016.00441)

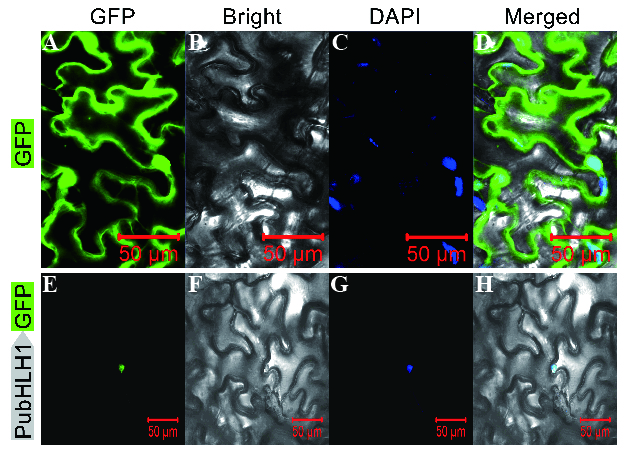

Supplement: Supplementary file 2 [file Image_1.JPEG]

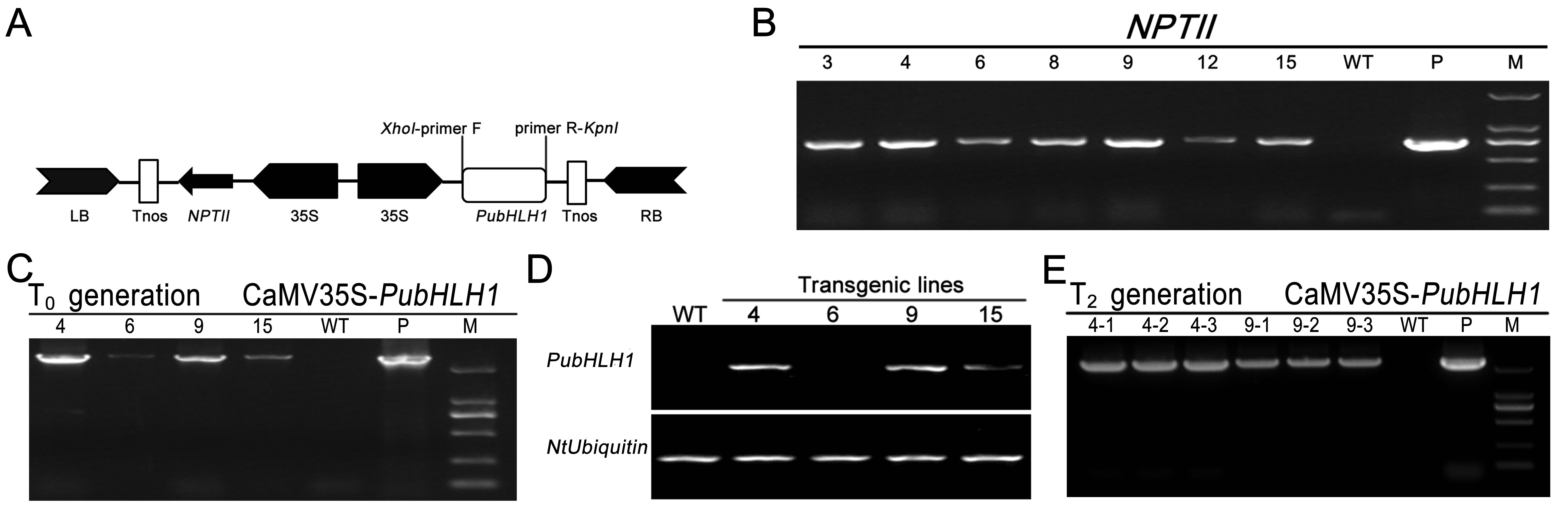

Supplement: Supplementary file 3 [file Image_2.JPEG]
